# Correcting for Superficial Bias in 7T Gradient Echo fMRI

**DOI:** 10.3389/fnins.2021.715549

**Published:** 2021-09-22

**Authors:** Pei Huang, Marta M. Correia, Catarina Rua, Christopher T. Rodgers, Richard N. Henson, Johan D. Carlin

**Affiliations:** ^1^Singapore Institute for Clinical Sciences, A^∗^STAR, Singapore, Singapore; ^2^MRC Cognition and Brain Sciences Unit, University of Cambridge, Cambridge, United Kingdom; ^3^Wolfson Brain Imaging Centre, University of Cambridge, Cambridge, United Kingdom

**Keywords:** 7T GE-fMRI, fMRI methods, superficial bias correction, Deming regression, computational modeling

## Abstract

The arrival of submillimeter ultra high-field fMRI makes it possible to compare activation profiles across cortical layers. However, the blood oxygenation level dependent (BOLD) signal measured by gradient echo (GE) fMRI is biased toward superficial layers of the cortex, which is a serious confound for laminar analysis. Several univariate and multivariate analysis methods have been proposed to correct this bias. We compare these methods using computational simulations of 7T fMRI data from regions of interest (ROI) during a visual attention paradigm. We also tested the methods on a pilot dataset of human 7T fMRI data. The simulations show that two methods–the ratio of ROI means across conditions and a novel application of Deming regression–offer the most robust correction for superficial bias. Deming regression has the additional advantage that it does not require that the conditions differ in their mean activation over voxels within an ROI. When applied to the pilot dataset, we observed strikingly different layer profiles when different attention metrics were used, but were unable to discern any differences in laminar attention across layers when Deming regression or ROI ratio was applied. Our simulations demonstrates that accurate correction of superficial bias is crucial to avoid drawing erroneous conclusions from laminar analyses of GE fMRI data, and this is affirmed by the results from our pilot 7T fMRI data.

## Introduction

Different layers in the neocortex support different types of neural computations. For instance, different layers of the visual cortex are preferentially involved in feedforward vs. feedback connectivity (Rockland and Pandya, 1979; Rockland, 2017), suggesting that they encode distinct “bottom-up” and “top-down” processes. However, because the cortical ribbon is only 2–3 mm thick in the sensory cortices (vonEconomo, 1929), conventional fMRI acquisitions with 2–3 mm isotropic voxels cannot resolve these layers (Dumoulin et al., 2017). Thus, most previous investigations of laminar organization involved invasive measurements that are generally unsuitable for human *in vivo* studies (Takahashi et al., 2016; Van Kerkoerle et al., 2017).

Recent advancement in MRI scanners, however, has changed the landscape. With higher field strengths (e.g., 7T), and in turn higher signal-to-noise ratios, human scanners are able to acquire data at submillimeter resolution, and thereby offer layer-specific or “laminar fMRI.” Recent 7T fMRI studies (Polimeni et al., 2010; Muckli et al., 2015; Kok et al., 2016; Lawrence et al., 2019) have suggested that top-down modulation of neural activity (for example, by attention or expectation) occurs in specific layers, though there is a disagreement about which layers. For example, Kok et al. (2016) used the Kanizsa triangle illusion to study attentional effects. By restricting their analysis to an regions of interest (ROI) that responded to the part of the visual field containing illusory contours but no actual stimulus input, they observed evidence of strongest top-down feedback effects in the activation of deep layers of visual cortex. Lawrence et al. (2019) utilized visual gratings and manipulated both bottom-up effects of visual contrast and top-down effects of spatial attention. While they found bottom-up effects on activation were strongest in the middle layer, their top-down effects were strongest in the superficial layers, contrary to Kok et al. (2016). Muckli et al. (2015) used partially occluded images, where part of a visual scene was replaced with a blank square. They again focused on an ROI that responded to the occluded region of the visual field, but rather than measuring the mean activation of that ROI, they attempted to decode the pattern of activity over all voxels within that ROI. While decoding of bottom-up information (in unoccluded images) was above chance and relatively constant across layers, they found that above-chance decoding of top-down information (in partially occluded images) only occurred in superficial layers. These discrepancies in layer selectivity could reflect a dissociation between superficial and deep layers in terms of the type of feedback they receive (depending on the precise paradigm), or could arise due to different analysis methods, which may result in different sensitivity to effects in particular layers. This highlights the importance of examining the assumptions behind the acquisition and analysis methods that researchers use.

In terms of acquisition, most laminar fMRI studies, including the ones cited in the above paragraph, have used gradient echo (GE) MRI sequences that are sensitive to the blood oxygenation level dependent (BOLD) signal (Olman and Yacoub, 2011), but alternative sequences are also increasingly popular (De Martino et al., 2013). The BOLD-GE sequences tend to provide good sensitivity to functional changes, but are also susceptible to signal artifacts, particularly from the large draining veins on the cortical surface (Boxerman et al., 1995; Rua et al., 2017).

An alternative class of sequences measure BOLD with spin echo (SE), or gradient and spin echo (GRASE) (Feinberg et al., 2015). These sequences are less prone to superficial bias but have lower functional contrast to noise ratios (Beckett et al., 2019) and are also limited by more stringent SAR constraints. A final class of sequences focuses on alternative metrics of neural activation. Cerebral blood volume (CBV) can be measured with vascular space occupancy (VASO) sequences (Lu et al., 2013; Huber et al., 2017b), and cerebral blood flow (CBF) can be measured using arterial spin labeling (ASL) sequences (Petcharunpaisan, 2010; Huber et al., 2017b; Kashyap et al., 2019). While these blood flow-based sequences are able to remove the spatial blurring due to draining veins, they come with drawbacks relative to GE-BOLD MRI. These drawbacks include the largest CBV changes being localized in the arteries and potential dilation retrogradely in the upper layers relative to the location of neuronal activation (Uludağ and Blinder, 2018). These methods also tend to have less sensitivity as a trade-off for their higher specificity (Huber et al., 2017b). Finally, VASO and ASL generally have longer TRs than GE and so are generally restricted to a smaller field of view (Huber et al., 2017a).

These limitations of alternative sequences have resulted in continued popularity for BOLD-GE laminar fMRI (Kok et al., 2016; Lawrence et al., 2019; de Hollander et al., 2020; Liu et al., 2020). However, the main drawback of using BOLD-GE sequences is the presence of superficial bias, where larger signals are observed in the superficial layers relative to the deep layers. Specifically, we are referring to the mitigation of variations in signal across layers at high resolution, and not the initial bias that arises due to large pial veins at low resolution fMRI. There are two neurological bases for the presence of this superficial bias: the presence of intra-cortical ascending draining veins and variations in baseline physiological parameters across cortical depths. Draining veins carry deoxygenated blood from the deep layers toward the superficial layers, which results in leakage effects across all voxels, whereby the measured signal is a combination of the laminar signal and unwanted signals from all the layers beneath (Markuerkiaga et al., 2016; Havlicek and Uludag, 2020). Secondly, variations in baseline CBV and relaxation parameters such as T2^∗^ across different cortical depths have a multiplicative effect on the signal (Kashyap et al., 2017) and thus, can also give rise to a superficial bias. The net effect is that BOLD-GE sequences have lower specificity and a bias toward higher signal in superficial layers, which complicates comparisons of task-related activation across layers.

There have been many different approaches to characterize and correct for this superficial bias, although few investigators have systematically explored whether different superficial bias-correction methods are expected to successfully correct bias under a particular model. In terms of bias models, Huber (2020) distinguishes three general classes of models: the linear-offset model, the multiplicative model and the leakage model. The multiplicative model seeks to emulate the effect of variations in baseline parameters across cortical depths, which has a multiplicative effect on the fMRI signal. Meanwhile, the leakage model attempts to capture the effect of draining veins on the fMRI data as it simulates the propagation of signal from the deep layers to the superficial layers, mimicking the effect of draining veins carrying oxygenated blood toward the superficial layers. In terms of methods that seek to correct bias in empirical studies, proposed solutions include Z-scoring timecourses (Lawrence et al., 2019), L2 normalization of beta estimates across conditions (Kay et al., 2019), taking the ratio of activations in two experimental conditions (Kashyap et al., 2017; Liu et al., 2020) and decoding using multi-voxel classification techniques (Muckli et al., 2015; de Hollander et al., 2020).

Here, we explore how seven of these superficial bias-correction methods perform under a simple multiplicative model applied to a visual attention paradigm. Specifically, we simulated data with a (non-linear) laminar profile, and compared how different superficial bias-correction methods perform in recovering these ground truth profiles in the context of a superficial bias nuisance effect. We also introduce a new application of Deming regression to this problem, and found that this method outperformed other commonly used approaches when conditions do not differ in regional-mean offset, while performing comparably to a region-mean ratio metric when conditions do differ. By introducing a method that provides robust correction for superficial bias, future studies will be able to benefit from the high sensitivity of BOLD-GE sequences for laminar fMRI.

## Materials and Methods

Since the simulations are matched to the design of the example dataset, we describe this experiment first. Processed simulation and fMRI data is available at https://github.com/MRC-CBU/LaminafMRIsimulations/tree/1.1. Full simulation and analysis code is also available at the same repository. Note that the main fMRI task is carried out at 7T while the localizer tasks were conducted at 3T.

### Experimental Design

**FIGURE 1:**
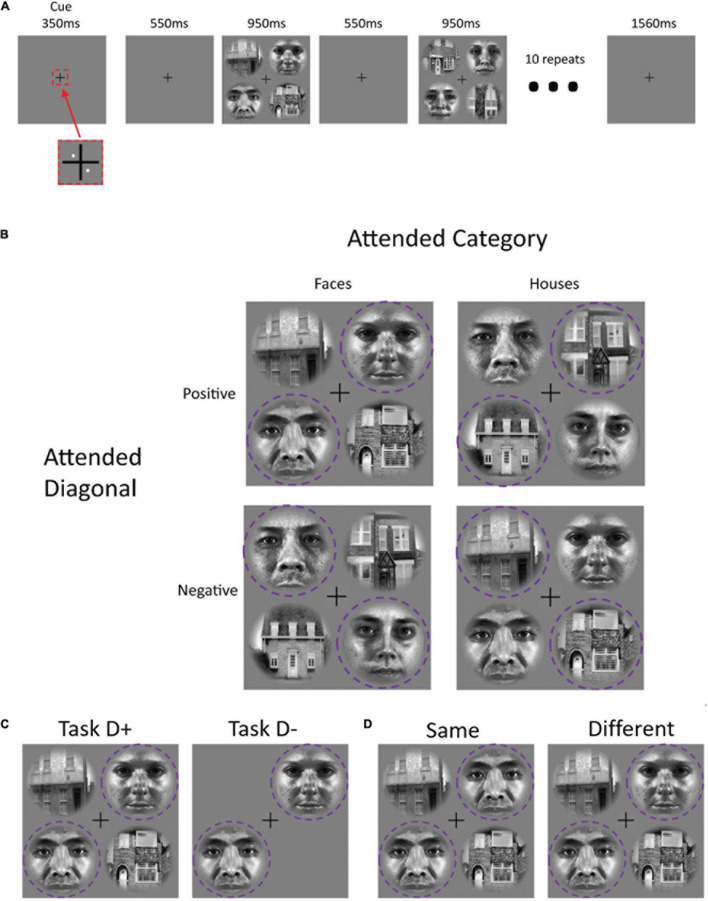
Panel **(A)** shows the experimental paradigm for each trial block. An initial pair of white dots cued participants to attend to a specific diagonal at the beginning of each task block. Ten image pairs from one category (faces or houses) appeared sequentially along the attended diagonal. Panel **(B)** illustrates the four main stimulus block types. In the “TaskD+” distractor-present condition, image pairs from the other category appeared along the opposite diagonal; in the “TaskD–” distractor-absent condition, no stimuli appeared in the opposite diagonal **(C)**. Participants decided whether the two stimuli on the attended diagonal were “same” or “different” **(D)**; 50% of trials involved the same stimuli. The purple dotted circle indicates the attended regions and was not present for the participant.

We designed a block-based sustained attention paradigm. On each trial, participants performed a same-different judgment of two images that were presented at diagonally offset locations around a central fixation cross (Figure 1). We varied the attended location (positive diagonal vs. negative diagonal of a 2 × 2 stimulus array around the fixation cross) and stimulus category (face or house images) between blocks of 10 trials. Each run comprised 20 blocks in total: attending to houses along the positive diagonal (H^45^), attending to houses along the negative diagonal (H^135^), attending to faces along the positive diagonal (F^45^) and attending to faces along the negative diagonal (F^135^). The conditions were presented in a sequence that was randomized separately for each run.

Between runs, we also manipulated the presence of a second pair of distractor image sequences, which, when present, were located at the opposite location and drawn from the opposite stimulus category. For both simulation and our fMRI acquisition, there were a total of eight runs: four with distractors present (TaskD+) and four with distractors absent (TaskD−). For the fMRI acquisition, we alternated the presence or absence of distractors between runs. In the simulation, each run is detrended individually and hence, there is no overarching effect due to the order of runs.

We attribute any selectivity for category or location when the distractor is present (TaskD+) to attentional selection, because both locations and both categories are present on each block, which provides complete matching of the conditions at the level of bottom-up stimulus-driven responses. By contrast, location and category selectivity during the no distractor (TaskD−) context could also reflect stimulus-driven effects of the stimulated visual quadrants or the presented object category. Some of the superficial bias-correction methods we explore below are based on the notion that the no distractor (TaskD−) context provides a control condition that can be used to correct any superficial bias in the estimates for attentional selectivity when the distractor is present (TaskD+). We will expand on this idea below.

#### Stimulus and Procedure

All stimuli were created using Matlab (2009a, The MathWorks, Natwick, MA, United States) and presented in the scanner using Presentation (v17.2). For the main experiment, the category stimuli were presented in a circular patch at four locations, diagonally from the fixation cross at 45, 135, 225, and 315°, respectively and spanning 0.16–2.42° eccentricity. The fixation cross was shifted up from the center of the screen by 2° visual angle due to visual obstruction of the lower segment of the screen by the head coil. There were a total of 20 face images and 20 house images for each category. All images were presented in grayscale and histogram-matched to equate luminance and root mean squared contrast. Thus, any category selectivity cannot be attributed to differences in brightness or contrast between the categories.

At the start of each block, two white dots (0.10° eccentricity) appeared for 350 ms indicating the pair of patches [either 45 and 225° (indicated by ^45^) or 135 and 315° (indicated by ^135^)] to which the participant should attend. This was followed by 550 ms of fixation. The stimuli then appeared for 950 ms, during which the participant was required to perform a same-different judgmenton the two attended stimuli, followed by 550 ms of fixation. The two stimuli were identical on 50% of trials. This stimulus-fixation trial was repeated 10 times within each block (Figure 1A). Between blocks, there was a rest block of fixation with a duration of 1560 ms (1260 ms for the two participants with TR = 2440 ms, see section “Data Acquisition” for more details). The duration was chosen to ensure that the start of each block was in sync with the start of a volume acquisition. In the distractor-absent condition, the display consisted of two stimuli from the attended category in the attended locations, while in the distractor-present condition, two additional stimuli from the other category appeared in the non-attended locations (Figure 1C).

#### Stimulus Design for Localizer (fMRI)

The localizer session comprised of four runs of a category-selective localizer.

The category-selective localizer task comprised 15-s blocked presentations of sequences of faces, scenes, objects, scrambled objects and fixation, with 1 s fixation between blocks. Each of these five block types appeared in a random order in each run. There were 20 blocks per run (four presentations of each block type). Within each block, 25 randomly selected stimuli from the current category were presented consecutively for 800 ms each. Participants performed a 1-back matching task while fixating on a black dot in the middle of the screen.

We used the categorical localizer data to define the following ROIs according to conventional functional criteria (see below for details): occipital face area (OFA), fusiform face area (FFA), scene-selective transverse occipital sulcus (TOS), and parahippocampal place area (PPA).

### Data Acquisition

Participants provided informed consent under a procedure approved by the institution’s local ethics committee (Cambridge Psychology Research Ethics Committee). A total of six healthy participants (two females, age range 20–41, two participants were authors of this study) were scanned for this pilot study.

Prior to the experiment, participants underwent a separate session of behavioral training with eye-tracking using an SMI high speed eye tracker. The participant attempted the same task and received feedback on their fixation levels after each run. We recorded calibrated eye position during each block, and measured fixation stability as the difference in standard deviation along the attended and neglected axes over the block. We repeated the task until this fixation stability metric met a criterion level of under 0.5° visual angle mean difference for two consecutive runs. As eye-tracking was not available in the 7T scanner, the behavioral training was important to ensure that participants were able to perform the task while maintaining fixation.

Participants contributed data over a total of two MRI sessions; one session of retinotopic and category localizers at 3T and one main experimental session at 7T. The 3T data were acquired on a Siemens 3T Prisma-Fit scanner using a standard 32-channel head coil, while the 7T data were acquired on a Siemens 7T Terra scanner using the Nova Medical 1Tx/32Rx head coil. At the start of each session, we also acquired a MPRAGE (3T) or MP2RAGE (7T) (Marques et al., 2010) structural that was used for co-registration across sessions. Participants maintained fixation on a cross in the middle of the screen throughout, and fixation accuracy was verified using the 50 Hz SMI MRI eye tracker system at 3T.

For 3T, the MPRAGE parameters were as follows: TR = 2,250 ms, TE = 3.02 ms, TI = 900 ms, GRAPPA = 2, FOV = 256 mm × 256 mm × 192 mm, Matrix size = 256 × 256 × 192, FA = 9°, ToA = ∼5 min and the EPI parameters for the functional localizer task were as follows: 3 mm isotropic voxels, TR = 2000 ms, TE = 30 ms, FA = 78°, Matrix size = 64 × 64 × 32, ToA = ∼11 min.

For the 7T data, the MP2RAGE parameters were as follows: TR = 4,300 ms, TE = 1.99 ms, TI 1 = 840 ms, TI 2 = 2370 ms, GRAPPA = 3, Matrix size = 320 × 320 × 224, FA 1 = 5°, FA 2 = 6° and the EPI parameters for the task-based fMRI were as follows: 0.8 mm isotropic voxels, TR = 2390 ms (2440 ms for two participants), TE = 24 ms (24.4 ms for two participants), FA = 80°, GRAPPA = 3, Matrix size = 200 × 168 × 84, ToA = ∼11 min. The TR and TE were slightly longer for two participants due to the peripheral nerve stimulation threshold being exceeded in the 7T scanner.

### Data Analysis

#### MRI Data Pre-processing

For the functional volumes acquired on the 3T scanner, the images underwent slice time correction and rigid body realignment using SPM12^1^. For the volumes acquired on the 7T scanner, the images first underwent slice time correction using SPM12, and then TOPUP (Andersson et al., 2003) in FSL v6.0 (Niazy et al., 2004) was applied to estimate the susceptibility-induced distortions, using the first five volumes of each experimental run plus five additional volumes acquired before each run with the reverse phase-encoding direction. The resultant distortion correction was applied to the entire run. Each fMRI volume was then individually realigned to the structural using boundary-based registration (BBR) (Greve and Fischl, 2009). In doing so, we use the structural as a reference to ensure that the volumes are co-registered with each other, which we previously showed to provide better realignment within and across runs for high-resolution fMRI data compared to standard methods that separate motion correction and co-registration (Huang et al., 2020).

Of note, some previous work has attempted to correct for superficial bias in this stage via the exclusion of voxels with low tSNR and high *t*-values on a fMRI statistical map (Olman et al., 2007; Jia et al., 2020; Zamboni et al., 2020). We attempted to exclude voxels with the bottom 30% tSNR in our analysis. However, this did not change our findings substantially (see Figure 7 and Supplementary Figure 1) and hence, was not included in the main analysis to reduce the number of potential confounds.

#### Regions of Interest

For the categorical ROIs, activation t-maps where obtained using SPM12 by fitting a GLM to the fMRI data from the categorical localizer runs. The activation maps were corrected for multiple comparisons using Gaussian random field theory in SPM12. The face-selective areas (FFA and OFA) were obtained from the t-score map from subtracting the object conditions from the face conditions. Similarly, the scene-selective areas (TOS and PPA) were obtained from the t-score map from subtracting the object conditions from the scene conditions. For each ROI, we identified the peak voxel in the expected anatomical location in a statistical map thresholded at *p* < 0.05 (uncorrected). We then grew the complete ROI by adding the contiguous voxel that was most selective for the localizing contrast iteratively until the ROI comprised 100 voxels (for implementation)^2^.

To improve the stability of the estimates and our sensitivity to any variations in the data across layers, we combined the TOS, PPA, OFA, and FFA regions to generate a pooled category-selective ROI. This approach is further justified in section “Laminar Analysis of Real 7T Data.” Note that we flipped the sign of the contrast vector to align with the region’s expected preference (e.g., F^45^+F^135^-H^45^-H^135^ for FFA and H^45^+H^135^-F^45^-F^135^ for PPA). All subsequent analyses concern this pooled category-selective ROI unless specifically noted otherwise.

We co-registered the 3T localizer session to the main experiment 7T session. This was done by first co-registering the 3T functional images to the 3T structural image using the SPM coreg function. The 3T structural image was then co-registered to the 7T structural image, again using the SPM coreg function. The transformations from both co-registration steps were then applied to the ROI mask images. No further transformation was necessary since the BBR realignment process realigns the functional 7T images to the 7T structural image. Note that the registration of the 3T localizer data to the 7T fMRI session results in an upsampling of the voxels from 3 mm isotropic resolution to 0.8 mm isotropic resolution to be congruent with the 7T fMRI data. This also results in the number of voxels in each ROI to increase from 100 voxels to an average of 2500 voxels.

#### Cortical-Depth Definition

The gray matter-white matter (GM-WM) and gray matter-cerebrospinal fluid (GM-CSF) boundaries were obtained from the FreeSurfer’s (version 6.0.0) reconstruction of each participant’s 7T structural image (Fischl et al., 2002). These boundaries were visually inspected to ensure that the segmentation was accurate. In cases of poor segmentation, the realignment between the structural and the FreeSurfer segmentation template was manually adjusted prior to repeating the FreeSurfer reconstruction. The boundaries were exported to CBStools (version 3.0.8) (Bazin et al., 2012) and used to generate three equivolume (Leprince et al., 2015) segmentations of the GM. Each GM voxel was then assigned to one of the three layers using a winner-takes-all approach, in which the voxel is assigned to the layer with which it has the largest overlap.

### Computational Simulations

Our computational simulations were carried out in Matlab (2019a, The MathWorks, Natwick, MA, United States); the code is available on github^3^.

For clarity, we describe our simulations in terms of categorical attentional selectivity for a face-selective area such as the FFA. However, the model is a general account for how fMRI responses arise as a function of neural modulations of interest and layer bias of no interest, so is applicable to other scenarios.

We model the effects of attention on neuronal responses, and how such modulations manifest in fMRI activations. In our model, the signal component in *N* = 2500 fMRI voxels is expressed as a sum over neuronal populations that are purely selective for houses or for faces. Neurons fire at rate *f* = 1 when their preferred category is present in the stimulus display, and *f* = 0 to the presence of the non-preferred category. We generate the number of neurons *n* that prefer each stimulus category for each voxel by sampling a half-normal distribution, and control category selectivity by varying the standard deviation of the distribution over categories. At the population level, for an ROI that prefers faces, we set the standard deviation of that distribution to 1.1 for faces and 0.5 for houses; for an un-selective ROI (see section “Simulating the Effects of No Region-Mean Preference”), we equate the standard deviations to 0.5 for faces and houses. To simulate inter-subject variability, the specific standard deviation used for each subject was sampled from a truncated Gaussian with mean equal to the above population values and standard deviation of 0.25 and values restricted between 0 and double that of the population value. Although selectivity at the voxel level in this formulation arises from differences in the relative frequency of face and house neurons, one could also interpret these standard deviation parameters as controlling the firing rates of equally sized populations.

In our model, attention operates as a scale factor *a* that increases the responses of neurons coding the attended category, while leaving the unattended category unaffected. We simulate layer differences in the strength of attentional modulation by varying this *a* parameter as a function of the layer *l*. The signal component of the response *R* at voxel *v* across all categories *c* can be expressed as


(1) $$R\left(v\right)=\sum_{c}f\left(c\right)\times n\left(v,c\right)\times a\left(c,l\left(v\right)\right)$$

where *l*(*v*) indicates the (dominant) layer captured by voxel *v*. We modeled the neural response *R(v)* for the duration of each block, based on the timings of conditions in our experimental paradigm, and convolved these responses with a canonical hemodynamic response function to generate a timeseries for each voxel, *h(v,t)*.

We added two sources of noise: A physiological noise source, which has a low-dimensional structure over voxels and which scales with layer depth, and a white thermal noise source, which is constant in magnitude over the simulated volume. The rationale for the physiological noise is to model known low-dimensional fMRI noise processes such as heartbeat, respiration and residual head motion (Liu, 2016), which voxels exhibit to different extents. Formally, the physiological noise, *E*_*p*_, was modeled by generating 20 independent Gaussian noise vectors for the entire timecourse and projecting a randomly weighted combination of the vectors onto each voxel (Huang et al., 2018). This physiological noise was scaled to match a random zero-mean Gaussian distribution with standard deviation of σ_*p*_. The resulting timeseries was then multiplied by a scalar *L*_*Bias*_ to capture the bias toward superficial layers. Lastly, we added thermal noise, *E*_*t*_, from Rician distribution (Gudbjartsson and Patz, 1995) with standard deviation of σ_*t*_, which is independent of superficial bias. This was done for all 2500 voxels to generate a simulated timecourse, *y(v,t)*, for voxel *v* in layer *l*. Thus:


(2) $$y\left(v,t\right)=L_{Bias}\left(l\left(v\right)\right)*\left(h\left(v,t\right)+E_{p}\left(v,t\right)\right)+E_{t}\left(v,t\right)$$

Having generated synthetic BOLD timeseries for each voxel, one can estimate the response to each condition by applying the general linear model (GLM), as is standard in fMRI analysis. To simulate the high-pass filtering commonly performed on real data to remove low-frequency noise in fMRI, first order sinusoidal and linear detrending was also performed. The GLM is based on the block timings and a canonical HRF, and produces a parameter estimate β for each condition and each voxel. The final contrast estimate (for a face-selective ROI) for the TaskD+ and TaskD− contrasts is then the subtraction of β for blocks when faces were unattended from that when blocks were attended.

To calculate the attentional contrast for our TaskD+ condition (when both positive and negative diagonals contain faces and houses, and the category being attended is equally often faces or houses), we take the simulated fMRI response during blocks where the participant is attending to the faces and subtract blocks where the participant is attending to the houses. The response for the TaskD+ contrast is therefore:


(3) $$R_{D}+\left(v\right)=\left(a\left(l\left(v\right)\right)\times n\left(v,f\right)+n\left(v,h\right)\right)-\left(n\left(v,f\right)+a\left(l\left(v\right)\right)\times n\left(v,h\right)\right)$$

where *f* = face, *h* = house, while in our TaskD− contrast, where faces or houses are only presented on the attended diagonal, the response is:


(4) $$R_{D-}\left(v\right)=a\left(l\left(v\right)\right)\times n\left(v,f\right)-a\left(l\left(v\right)\right)\times n\left(v,h\right)$$

**FIGURE 2:**
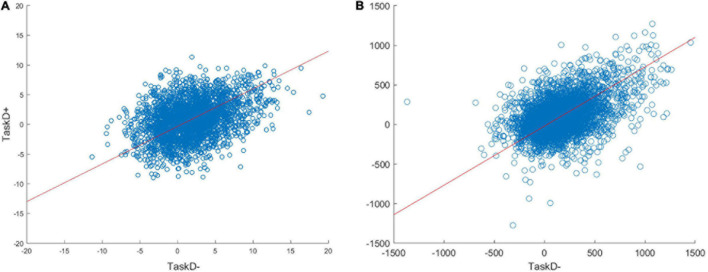
Scatterplots of the voxel responses (contrast estimates from the GLM) to TaskD+ against TaskD– comparing the simulated data with *a* = 3 **(A)** against the real 7T data **(B)**. The scatterplots look similar, suggesting that the simulation is capturing the behavior of real voxels. Note that the methods we evaluate are invariant to the absolute scale of the response, so these do not require matching; rather, we are only interested in the relation of the values across task types, i.e., the gradient of the best fit line (indicated by the red line), which is comparable across simulated (0.65) and real (0.72) data.

We explored a wide range of physiological and thermal noise parameters, and the final values of σ_*p*_ = 11 and σ_*t*_ = 15 were chosen by visually comparing scatterplots of TaskD+ against TaskD− for our simulated dataset against the experimental fMRI dataset for the pooled ROI (Figure 2). Furthermore, the combination of noise parameters and standard deviation values for the half-normal distributions were chosen such that the distribution of t-statistic for the simulated contrast was similar to the experimental dataset (see Supplementary Figure 2). Similarly, an attentional modulation factor of *a* = 3 was used to reflect the estimate generated from the experimental fMRI dataset. To simulate weaker attentional modulation in the middle layer, an attentional modulation factor of *a* = 2 was used for the middle layers. Note that while the exact parameter values were coarsely estimated, the results of our simulation hold across a wide range of parameters (Supplementary Figure 3) and are thus not restricted to the specific values used in this simulation.

The seven attentional modulation metrics were evaluated using simulated datasets with a V-shaped attentional modulation profile across layers of *a*(*l* = 1…3) = [323]. This profile indicates stronger attentional modulation in the superficial and deep layers, as might be expected from stronger feedback connectivity in these layers (Rockland and Pandya, 1979; Rockland, 2017). We repeated the simulation 10,000 times to calculate the accuracy and reliability of the seven attentional modulation metrics.

### Attentional Modulation Metrics

We were interested in validating the accuracy and reliability of the following seven metrics of attentional modulation, which were applied to both the simulated and real data. The metrics were analyzed in two ways. Firstly, the variation of each metric across layers was plotted and compared to the “ground truth” attentional modulation graph. Secondly, for each metric, we quantified the contributions of the superficial bias and attentional modulation toward the laminar profile. This was achieved by correlating the simulated data with either a [1 0 −1] contrast vector (for superficial bias) or [0.5 −1 0.5] (for attentional modulation) to determine the contributions of the two factors. The ideal laminar profile correlates with attentional modulation and not with superficial bias.

#### Ratio Metrics

The first group of attentional modulation metrics can be classified as ratio metrics; these metrics take the ratio between two experimental conditions (in this case, TaskD+ and TaskD−) to remove the superficial bias across layers. This method is motivated by the assumption that the baseline parameters, including baseline blood volume, oxygen extraction fraction, T2^∗^ relaxation time and echo time, have a multiplicative effect on BOLD sensitivity, as suggested in Kashyap et al. (2017) and conforms to the single vascular compartment BOLD signal models (such as in Buxton et al., 2004). Thus, we can formulate the BOLD signal change as δ*S* = *L(l)* × *R*, where *L* is a function of the baseline physiological parameters that influence the BOLD response and *R* is the actual change in CBV and concentration of deoxygenated hemoglobin in response to neural activity. In our simulation, L_bias_(l) is used to model L(l). We assume that *L(l)* is constant within each cortical layer but varies across cortical layers, while *R* is the quantity of interest. Thus, by taking a ratio of the signal changes for two contrast estimates for each voxel, we can remove the dependence of the contrast on the baseline parameters:

$$\frac{\delta S_{1}}{\delta S_{2}}=\frac{L\left(l\right)*R_{1}}{L\left(l\right)*R_{2}}=\frac{R_{1}}{R_{2}}$$

Note that while the attentional modulation, *a*, is defined to be greater than 1, the ratio metric of TaskD+/TaskD− in our simulation and experiment should always be less than 1. We refer to this ratio metric of TaskD+/TaskD− as selectivity, *S*, to differentiate it from the attentional modulation. The selectivity looks at the magnitude of top-down attentional modulation in TaskD+ as a fraction of both top-down attentional modulation and bottom-up stimulus activation in TaskD−. If we assume that our model is accurate, the relationship between selectivity and attentional modulation is:

$$S=1-\frac{1}{a}$$

##### Ratio of individual voxels (voxel ratio)

Given the above argument, one possible approach is to calculate the ratio of two values the GLM contrast for the TaskD+ condition relative to that for the TaskD− condition, for each voxel separately, and then average these ratios across voxels in a layer:

$$\frac{1}{n_{v}}\sum_{N_{v}}\frac{TaskD+}{TaskD-}$$

We refer to this metric as the “voxel ratio” throughout. However, this method has a high sensitivity to voxels where the TaskD− contrast is close to zero (e.g., because no attentional effect, or owing to random noise or fMRI susceptibility artifacts), which can produce extreme outliers. Assuming both TaskD+ and TaskD− contrasts follow a Gaussian distribution, the resultant voxel ratio would have a heavy-tailed distribution, on which statistical tests are difficult. As such, we do not expect the voxel ratio to generate stable estimates but include it for completeness.

##### Ratio of entire ROI (ROI ratio)

To reduce the effect of outlier voxel values, one can first average (or sum) responses across voxels in a layer, before taking the ratio of the averages in each condition (cf. Liu et al., 2020):

$$\frac{\sum_{N_{v}}TaskD+}{\sum_{N_{v}}TaskD-}$$

We refer to this metric as the “ROI ratio” for simplicity. A similar approach was adopted by Kashyap et al. (2017), where they compared the average of the activation peak against the average post-stimulus undershoot. However, by taking the average prior to the ratio, this method discards any pattern information that might be present within the voxels (we return to this point below).

##### Deming regression

Here, we chose not to average across voxels, but to regress one condition against the other condition and estimate the gradient of the best fit line. Importantly, we used Deming regression to estimate this gradient, rather than ordinary least squares, because Deming regression accounts for errors in both variables (Adcock, 1878). We theorize that this gradient would provide a stable estimate of the selectivity. A key benefit of Deming regression is that it does not require a regional-mean activation level difference between the two conditions, unlike the ROI ratio approach above. This makes the method potentially attractive for cases where voxels within an ROI have strong but opposing selectivity, as might for instance be expected in retinotopic maps of the visual field.

In our study, we assume the errors in both variables have similar variances (i.e., δ = 1) as data for both TaskD− and TaskD+ were acquired during the same scanner session and in an interleaved manner across runs. Thus, we can assume that the variance of the noise is similar across TaskD+ and TaskD−. Note that this special case of Deming regression can also be referred to as Orthogonal regression.

#### Z-Scoring of Data

Rather than taking ratios or gradients of the estimated responses (i.e., contrasts of GLM Betas), another approach is to normalize the original data (*y(v,t)* in Eq. 2) before fitting the GLM, for example by using Z-scoring (Lawrence et al., 2019), in an attempt to equate the scaling across layers. Here, after Z-scoring the data and fitting the GLM, we simply calculated the contrast estimate for the TaskD+ condition. Z-scoring assumes that the noise and the signal of interest have a linear correlation. However, given that this is not the case in our simulation (physiological noise scales with the signal but thermal noise does not), we expect Z-scoring to perform worse than the ratio approaches.

#### L2 Normalization

The last method included in our analysis is L2 normalization, as suggested in Kay et al. (2019). Similar to Z-scoring, this method seeks to eliminate the multiplicative effect of superficial bias by normalizing the response. However, the normalization (scaling factor) is estimated across parameter estimates (from a GLM fit) rather than across scans (i.e., the original timeseries, as in Z-scoring). The scaling factor (the square root of the sum of squared values) is also relative to zero, which depends on the baseline in the GLM, rather than being relative to the mean, as in Z-scoring.

#### Multivoxel Pattern Analysis

The methods described above that first average over voxels within an ROI are only able to estimate a layer’s univariate activation. An alternative approach is to examine the multivariate information across voxels, e.g., by comparing the ability to classify whether faces or houses were attended. This was the approach taken by Muckli et al. (2015). The logic underlying this approach is that cross-validation performance will be unaffected by superficial bias as long as the effect of interest and the noise scale together with overall signal magnitude. Like Z-scoring above, any additive (thermal) noise components will invalidate this assumption and reintroduce sensitivity to layer depth.

Here, we compared the ability of two methods of classification, support vector machines (SVMs) classification and linear discriminant contrast (LDC) to discern between attending to houses vs. faces in the TaskD+ condition. SVM is a robust method that has been widely used in fMRI analysis (Hoeft et al., 2011; Meier et al., 2012; Weygandt et al., 2012; Abdulkadir et al., 2013) and have also been used previously for laminar analysis (Muckli et al., 2015). LDC is often preferable for fMRI decoding since it is a continuous measure that does not exhibit ceiling effects (Zhu et al., 2008; Misaki et al., 2010; Yoon et al., 2012; Kriegeskorte and Diedrichsen, 2016; Huang et al., 2018). Furthermore, unlike SVM, it does not require multiple instances for effective training, and therefore avoids issues to do with small numbers and temporal autocorrelation of single-trial/block estimates from the same run. Previous work demonstrated that LDC outperforms SVM in terms of detecting a difference in discriminability (Huang et al., 2018) and in terms of overall reproducibility of pairwise discriminants over splits in the data (Walther et al., 2016). Both SVM and LDC used leave-one-out cross-validation across the four runs of each task.

##### SVM

We used the “fitcsvm” classifier in the Matlab Bioinformatics toolbox, with default settings (specifically, a linear kernel with C = 1). To obtain multiple patterns for training, each block within a run was modeled with a separate regressor in a new GLM, producing 60 block estimates for training and 20 for testing on each fold of leave-one-run-out cross-validation. The final result is mean classification accuracy (% of test blocks correct).

##### LDC

Linear discriminant contrast is a continuous statistic related to Fisher’s linear discriminant. First, the training data is used to generate a set of weights to maximize the distance between the conditions of interest (e.g., houses vs. faces). This set of weights is referred to as the discriminant. The LDC quantifies the difference between the two conditions in the testing data, measured on this discriminant.

As LDC does not require multiple training patterns, all blocks of the same type (within the training set) were modeled with a single regressor in the GLM. The contrast between attending to faces vs. houses generates a distance metric, which is normalized using the sparse covariance matrix of the noise residuals (Ledoit and Wolf, 2003) to produce a weights vector. The dot product of the weights vector with the pairwise contrast estimate from the held-out test run produces the LDC test statistic. The final result is the average LDC across the four folds of leave-one-out cross validation runs and normalized by the square root of the number of voxels (which differed by ROI in the real data).

## Results

### Computational Simulations

#### Comparison of the Seven Attentional Modulation Metrics

**FIGURE 3:**
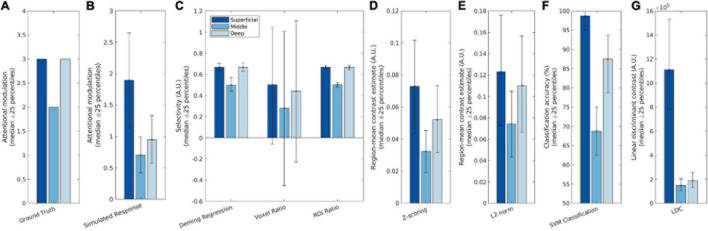
Simulation results showing **(A)** the ground truth (neural response), **(B)** the simulated measured (BOLD) response, and **(C–G)** the seven attention metrics. Deming regression, Voxel ratio and ROI ratio were plotted on the same scale as they are commensurate; the other four metrics have different scales so were plotted individually. The different bar colors represent the different layers, with the dark blue representing the superficial layer and the light blue representing the deep layer. The bars indicate median performances over iterations of the simulation, while the error bars indicate the 25th and 75th percentile values.

For our main simulations, we simulated an attentional modulation that is strongest in the superficial and deep layers and weaker in the middle layer. The simulated fMRI responses were then measured according to the seven different metrics and compared against the ground truth profile to verify their accuracy and precision. Selectivity estimates using Deming regression, Voxel ratio and ROI ratio were plotted on the same axis as they generate commensurate estimates; the remaining three metrics were plotted on individual axes and scaled to best match the ground truth profile. In addition, we also plotted the raw contrast estimates and “ground truth” attentional modulation as a baseline comparison. The “ground truth” attentional modulation (Figure 3A) is obtained from the attentional modulation [a(c,l(v))] that is used as a model input while the raw contrast estimates (Figure 3B) is given by R_D_+_(v) in Eq. 4 and reflects the response obtained by fitting a GLM to the simulated data and contrasting the blocks, identical to what is done to the real fMRI data in section “Laminar Analysis of Real 7T Data.”

As can be seen in Figure 3, both Deming regression and ROI ratio were able to replicate the attentional modulation profile with high precision: The variability across iterations was low, as the percentile error bars illustrate. Though the voxel ratio recovered the V-shaped profile, it had very large error bars, owing to extremely high values for some voxels with a denominator (TaskD−) close to zero.

Z-scoring the data before fitting the GLM produced a slanted V profile, indicating contributions from superficial bias as well as attentional modulation. L2 normalization performed a bit better, being able to retain the V shaped profile, but still showing a significant difference between the deep and superficial layers due to superficial bias. Furthermore, the L2 normalization is also substantially noisier than both Deming and ROI ratio metrics.

The SVM and LDC multivoxel classification methods produced similar “slanted V” profiles, with residual effects of superficial bias. The LDC method exhibited particularly dramatic superficial bias, with strong discriminant values for the superficial layer where the response magnitude was greatest.

#### Quantifying the Relative Contributions of Superficial Bias and Attentional Modulation to Laminar Profile

We correlated the laminar profile of each metric (normalized by their mean) with either a [1 0 −1] or a [0.5 −1 0.5] vector to obtain a summary measure of the contributions of superficial bias and attentional modulation across layers. This was done by multiplying the estimates of each layer with the corresponding vector element and summing the result. These summary measures indicate the reliability of any apparent layer differences and provide a means to compare the magnitude of attentional modulation and superficial bias effects within each metric.

**FIGURE 4:**
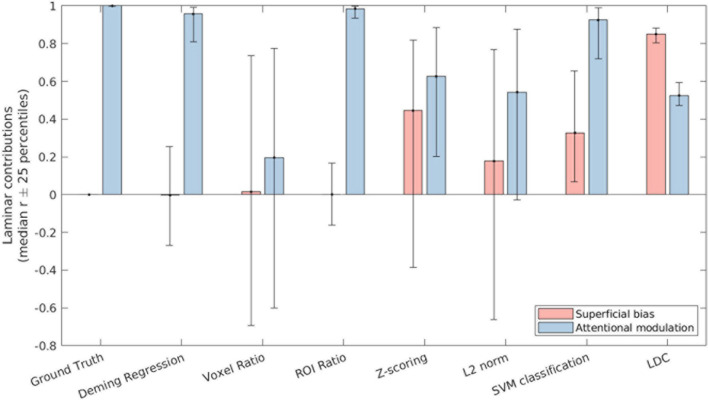
The contribution of superficial bias (pink bars) and attentional modulation (blue bars) to the laminar profile as recovered by the respective attentional metrics. The bars indicate the median correlation over iterations of the simulation, while the error bars indicate the 25th and 75th percentile values.

As shown in Figure 4, both Deming regression and ROI ratio were highly correlated with the ground-truth attentional modulation effect, with almost zero contribution of superficial bias. Both metrics also exhibited low variability over iterations, as indicated by the 25-percentile error bars. By contrast, the voxel ratio was highly variable. While relatively stable over iterations, the Z-scoring metric was substantially influenced by the nuisance superficial bias effect. This superficial bias was less pronounced after L2 normalization, but still present. Similar effects were observed for the SVM metric. The LDC metric had the lowest variability over iterations, but exhibited the strongest influence of superficial bias out of all evaluated metrics.

The SVM and LDC metrics exhibited distinct effects even though both are based on linear discriminants. We reasoned that this could reflect compressive effects of close-to-ceiling performance for the SVM (Figure 3F). This could obscure a superficial bias effect since the highest accuracy is in the superficial layer, where the bias is strongest. As a continuous metric, LDC does not exhibit ceiling effect. This can be demonstrated by repeating the simulation with increased noise levels (such as σ_*p*_ = 20 and σ_*t*_ = 30, Supplementary Figure 4), under which the profiles for the SVM and LDC metrics were comparable, confirming that the apparent differences between the SVM and LDC metrics in Figures 3, 4 reflect an SVM ceiling effect.

#### Simulating the Effects of No Region-Mean Preference

**FIGURE 5:**
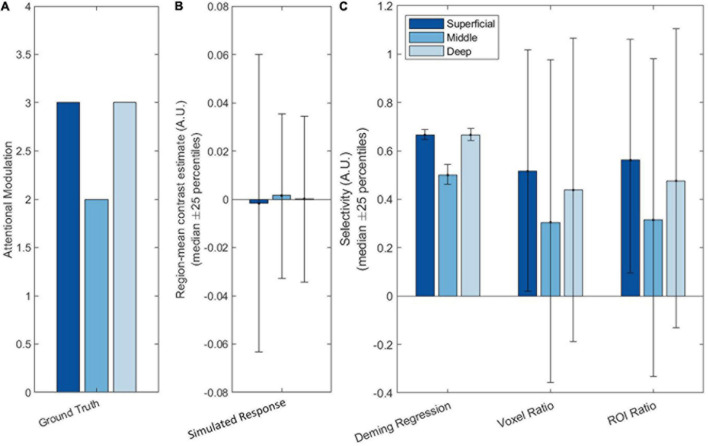
Simulation results showing **(A)** the ground truth (neural response), **(B)** the simulated measured (BOLD) response, and **(C)** Deming regression, Voxel ratio and ROI ratio in the absence of a global preference. The different bar colors represent the different layers, with the dark blue representing the superficial layer and the light blue representing the deep layer. The bars indicate the median effect over iterations of the simulation, while the error bars indicate the 25th and 75th percentile values.

Both the Deming regression and ROI Ratio showed similar results in our initial simulations. However, Deming regression is potentially sensitive to local variations within an ROI, even if there is no global preference of that ROI. To illustrate this, we repeated the simulation with the same underlying [3 2 3] attentional modulation (Figure 5A) but removed the global preference for houses by sampling the density of house responsive cells and face responsive cells from the same distribution (folded normal distribution with mean 0 and standard deviation 0.7). Thus, each voxel has an equal chance of preferring faces or houses, with a resultant near-zero global preference for either house or faces (on average across simulations). As Figure 5B shows, there was no distinguishable variation in the measured response. However, Figure 5C shows that only Deming regression was able to recover the V-shape with sufficient sensitivity (i.e., relative to the error bars).

It is interesting to note that, while the ROI ratio (like the Voxel ratio) is extremely noisy when there is no global preference, there is still a hint of a V-shape in the means in Figure 5C. We suspect this occurs because, while the overall preference averages to zero across all simulations, by chance, most individual simulations still have some global preference for either faces or houses. These small, but persistent, global preferences would still be sufficient for a ratio metric to recover the attentional modulation to some extent. However, as the percentile error bars illustrate, these ratio metrics are likely to be too variable to be useful when global preferences are near zero. This result demonstrates that Deming regression is the only method that can robustly detect layer specific modulations even in the absence of region-average differences.

### Laminar Analysis of Real 7T Data

**FIGURE 6:**
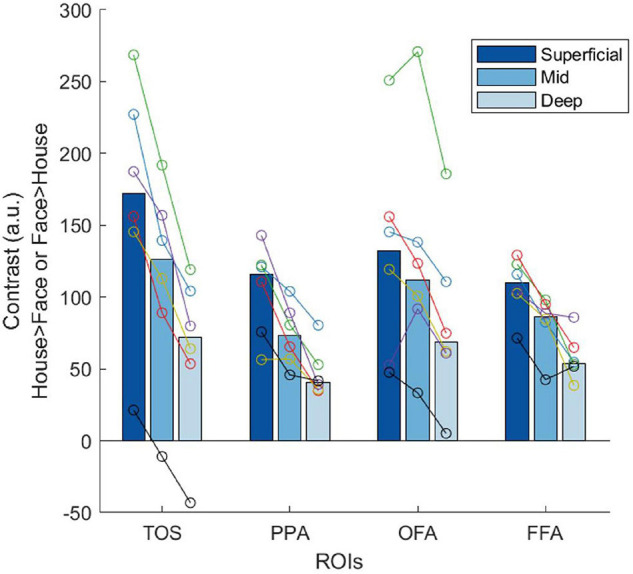
Plots of the contrast estimates obtained at 7T across different layers for the four different ROIs in the TaskD+ condition. The bars represent the median of all six participants, while each color in the overlaid circles represent an individual participant. The same color represents the same participant throughout all plots.

**FIGURE 7:**
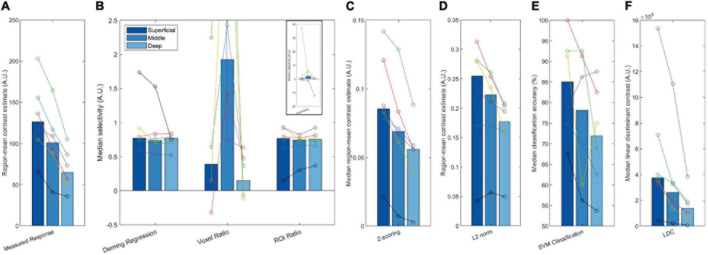
Plots of the measured response **(A)** and attentional modulation metrics (Deming regression, Voxel ratio, ROI ratio, Z-scoring, L2 norm, SVM classification, and LDC, **B–F**) across different layers. These bars represent the median of all six participants, with each set of joint circles represent an individual subject. The same color represents the same participant throughout all plots. The axis range of panel **(B)** has been restricted due to extreme outliers for the voxel ratio. The full range of this data is shown in the panel inset.

Initial laminar analysis of the 7T data showed strong selectivity in the superficial layers with a constant decrease toward deeper layers, consistent across all ROIs. In Figure 6, we plotted the contrast estimates for the TaskD+ condition for the ROIs to illustrate the consistency of the superficial bias profile across the ROIs. For the scene-selective TOS and PPA, the contrast estimate is defined as taking the house attention condition and subtracting the face attention condition, while for the face-selective OFA and FFA, the contrast estimate is defined as taking the face attention condition and subtracting the house attention condition. This definition is chosen to align with the category preference of the ROI so as to generate a positive contrast for all ROIs. This reduction in selectivity with layer depth is consistent with previous evidence for a superficial bias in GE sequences (Polimeni et al., 2010; De Martino et al., 2013). In order to obtain maximally robust estimators for the following analyses, we pooled the voxels from TOS, PPA, OFA, and FFA into a single pooled “category-selective” ROI. This step can be justified by the considerable variability across the six participants in the context of relatively higher similarity of selectivity profiles across the four ROIs. The pooled ROI exhibited substantially reduced between-participant variability (Figure 7A), while preserving the superficial bias profile we observed in the individual ROIs. In the following analyses, we focus on this pooled ROI, since we had no hypotheses concerning regional specificity of these effects. Furthermore, repeating the analysis on individual ROIs (Supplementary Figure 4) yielded similar, but substantially noisier, results as compared to the pooled analysis (Figure 7).

#### Laminar Analysis of 7T Data Using Attentional Modulation Metrics

The attentional modulation of the voxels as estimated with Deming regression was constant across layers (Figure 7B). Considered together with the simulation results, this indicates that the increased selectivity in superficial layers that we observed in Figure 6 can be explained by GE superficial bias. The ROI ratio metric demonstrated a similar lack of variation across layers, and comparable inter-subject variability. The voxel ratio was highly unstable across participants (see inset panel for full data range). The remaining four measures (Z-scoring, L2 normalization, SVM classification and LDC) exhibited stronger effect in superficial layers (Figures 7C–F), consistent with the sensitivity to superficial bias that we observed in our simulations.

Taken together, our results suggest that the superficial bias, as shown by the decrease in measured response across layers (Figure 7A), is successfully removed by both Deming regression and ROI ratio metrics (Figure 7B), giving rise to the flat selectivity profiles. Other methods were unsuccessful in removing the superficial bias.

## Discussion

A key challenge for interpreting layer analysis of high-resolution GE EPI fMRI data is that signal magnitude decreases with layer depth. This study investigates methods for correcting such superficial bias effects. We used computational simulations to evaluate the ability of seven different metrics to recover an attentional modulation layer effect of interest in the presence of a superficial bias nuisance effect. Two of the evaluated methods were proposed by us (Deming regression, LDC) and the remaining have been previously used in the literature (Voxel and ROI Ratio, Z-scoring, L2 normalization, and SVM). Only the ROI Ratio and Deming Regression metrics were able to recover the attentional modulation layer effect accurately and precisely. While the remaining metrics were able to detect some underlying differences in attentional modulation across layers, they were either noisier than the aforementioned two metrics (in the case of the Voxel Ratio) or retained a substantial component of residual superficial bias (in the case of Z-scoring and L2 normalization, or the multivoxel methods of SVM and LDC). Such partial correction for layer bias is particularly concerning because it might lead to mistaken inferences. Thus, the main contribution of our study is to demonstrate that Deming regression and ROI ratio are the most promising metrics for layer analysis of GE fMRI data.

Although the Deming regression and ROI ratio metrics performed similarly in the context of a region-mean activation-level difference between the conditions, we found that Deming Regression was better able to extract layer-profiles when we simulated a scenario where activation-level differences were near zero. This property may be useful in early visual areas such as V1, where an ROI might not show a global preference for two orientations of a visual stimulus, even though voxels within that ROI often show a local preference for one or other orientation (Kamitani and Tong, 2005; Alink et al., 2013). Conversely, our simulations suggest that the ROI ratio metric produces slightly less variable estimates when ROIs do exhibit strong activation-level differences. In summary, we recommend Deming regression as a general solution for correcting superficial bias in GE fMRI, although studies focused exclusively on effects carried by the regional mean may realize a small improvement by adopting the ROI ratio metric instead.

The substantial residual superficial bias in the Z-scoring, L2 normalization, SVM and LDC metrics can be explained by the characteristics of fMRI noise, which likely exhibit components that are both additive and multiplicative with respect to layer depth in GE fMRI data. For instance, Z-scoring assumes a linear relationship between the noise and the contrast of interest, and normalizes the contrast by dividing it by an estimate of noise from the variance in the data. However, since our model includes both thermal (laminar invariant) and physiological (laminar dependent) noise sources, the variance in the data is not a perfect reflection of the superficial bias. Thus, Z-scoring is unable to fully correct the superficial bias in our simulations. Similarly, L2 normalization assumes that averaging the voxel response across time can accurately capture the superficial bias, an assumption that would only hold true without additive noise. Finally, multivariate pattern analysis methods (SVM and LDC) fail to account for the superficial bias in the data because relatively greater contrast to noise ratios (due to superficial bias) leads to more robust contrast and better classification/LDC values. These metrics are only expected to correct layer bias successfully if all noise components scale with layer depth; a noise model that would be implausible for high-resolution fMRI where substantial additive thermal noise is inevitable.

Interestingly, LDC and SVM showed different sensitivities to superficial bias vs. attentional modulation in the simulations (Figure 4). As we have observed previously (Huang et al., 2018), LDC results had smaller variability across iterations, reflecting greater stability in the estimates. However, LDC also demonstrated higher sensitivity to the laminar bias. We believe that this is primarily caused by the performance of the SVM classifier being close to 100% in the superficial layer. This ceiling effect results in a non-linear relationship between the functional activation and classification accuracy and partially masks the superficial bias. By contrast, LDC is expected to scale linearly with response magnitude (Arbuckle et al., 2019), which in this context means it is better able to capture the full extent of the superficial bias. A repeated analysis with higher noise levels confirmed that SVM and LDC metrics performed more similarly when the SVM classifier’s performance was brought down from ceiling levels.

Our numerical simulations (see text footnote 3) may be useful to test yet other approaches to correcting for superficial bias. Nonetheless, the code makes several simplifying assumptions for ease of calculation and generalization, which we list here for clarity. Firstly, we assumed that neurons are purely responsive to faces or houses only, and that the voxel BOLD response is a simple sum of populations of face- and house-cells within the voxel. Secondly, we assumed that attention modulates responsive cells only by applying a gain factor and there is no constant additive component of attention. Thirdly, superficial bias was modeled as a gain factor on responses. Fourthly, we only modeled two sources of noise: at the level of the true hemodynamic response and at the level of measurement of that response, with only the former noise term being scaled by the laminar bias. Moreover, while we introduced some spatial covariance in this noise across voxels (which could reflect, for example, effects of head motion), we did not explore the degree of this spatial covariance, nor explore effects of the temporal autocorrelation known to exist in fMRI data (e.g., from physiological noise sources). While it is not obvious that changes in these assumptions would affect the current conclusions, this may be worth exploring in future simulations. Note also that we set the parameters of the simulation to approximately match the ratio of activations in the two conditions from the voxels in real 7T data. Supplementary Figure 3 shows that the same pattern of results emerges across a range of values for the signal and noise within voxels. One might also wonder whether the results depend on the number of voxels, e.g., for smaller ROIs. When we explored a range 2500 down to 100 voxels, we did not observe any differences in the relative performance of the metrics (only the expected increase in overall variance for all metrics). Thus while other parameter settings could produce different results, we think that it is unlikely that the other four metrics would outperform ROI ratio and Deming regression within the range of realistic parameters.

A final key limitation of our numerical simulations is that they are based on a multiplicative scaling model of superficial bias (Kashyap et al., 2017; Huber, 2020). While we believe this is more plausible than the linear-offset model discussed by Huber in the above reference, there are more sophisticated models that consider leakage effects (Markuerkiaga et al., 2016; Havlicek and Uludag, 2020). Based on our anatomical understanding of the basis for superficial bias, it is likely that a more complex model consisting of both multiplicative effects (variations in BOLD response parameters across layers) and leakage effects (presence of draining veins across layers) is needed to fully capture the intricacies of the superficial bias. However, the implementation of such a model, specifically the leakage effects, would require making specific assumptions of the vascular architecture that are likely to vary across different ROIs, and are not universally applicable. Moreover, modeling the leakage effects requires substantially more parameters to be defined, such as the radius of ascending veins, volume of capillaries, baseline intravascular and extravascular relaxation rate and their signal ratio, relative amounts of CBV in the microvasculature and ascending veins, among many others. These parameters either have to be obtained experimentally through additional measurements, or assumed using *a priori* knowledge of the vascular structure and associated parameters. In contrast, the multiplicative model, and in turn the ratio and Deming Regression methods, relies on fewer assumptions, and is likely to generalize across a variety of brain regions. This simple multiplicative model can serve as a preliminary filter: methods that fail to account for the multiplicative effect of superficial bias is unlikely to perform well in a more complex model with both multiplicative and leakage effects, even if they successfully account for leakage effects.

While more complicated models and methods may be necessary to extract laminar profile for responses of a single condition vs. baseline, we suspect that when the interest is in the difference between two or more conditions, taking an ROI ratio or estimating the slope of a Deming Regression is a simpler and effective way of removing the scaling component of the superficial bias effect. However it must be kept in mind that leakage effects might not be corrected by these methods as they are not simulated in the model. This limitation is particularly pressing when differences between layers are not found, since this can potentially reflect leakage.

We also applied the seven correction methods to the GE-fMRI 7T data acquired on the same paradigm. The uncorrected data showed the characteristic increase in BOLD signal toward superficial layers (Kok et al., 2016; de Hollander et al., 2020). This superficial profile remained after Z-scoring, L2 normalization and applying SVM or LDC, while the Voxel Ratio produced an extremely variable estimate, suggestive of greatest modulation in the middle layer. However, the Deming Regression or ROI Ratio metrics produced a largely flat profile across layers, with low variability across participants compared to the other metrics. These results are broadly consistent with the numerical simulations, and suggest that apparent modulations in selectivity across layers in the initial regional-mean contrasts can be explained in terms of superficial bias in the GE-fMRI signal, rather than a difference in attentional modulation as such. However, the small sample size of the initial study reported here prevents us from drawing strong inferences about the magnitude of layer-specific effects of attention in the population.

Our failure to observe a layer-specific effect of attention contrasts with other findings using similar manipulations of “top-down” processes (Muckli et al., 2015; Kok et al., 2016; Lawrence et al., 2019). These studies are quite heterogeneous in experimental design and have yet to be independently replicated, so completely consistent findings may not be expected. However, we note that some of these results were obtained using metrics that our numerical simulations suggest do not successfully correct superficial bias, which may provide a further explanation for any discrepancies. In particular, we believe that inadequate correction for superficial bias may explain the superficial profile reported by Lawrence et al. (2019) for spatial attention with a Z-scoring metric, and the superficial profile reported by Muckli et al. (2015) for SVM classification of visual content outside the voxelwise receptive field. Thus, it would be difficult to draw any conclusions as to the presence of any neuronal response differences across layers based on those results. By contrast, Kok et al. (2016) reported relatively stronger selectivity for illusory contours in deep layers, a result that runs opposite to the expected direction of a superficial bias effect. Such a pattern is not expected by superficial bias–instead the selectivity estimates in Kok et al. (2016) would likely be stronger after correction for superficial bias. In general, we urge caution in interpreting GE fMRI layer analyses where superficial bias has not been appropriately corrected.

For completeness, we would also like to highlight that this is a rapidly growing field and newer methodologies, such as phase regression (Stanley et al., 2021), are constantly emerging to address the problem of superficial bias in GE fMRI. In their paper, Stanley et al. (2021) utilized the phase differences to estimate the contribution from vessels, and were able to generate similar laminar profiles to those observed using SE fMRI.

## Conclusion

In this study, we demonstrate that Deming regression and ROI ratio can adjust for superficial bias across cortical layers. This is important because there is a growing wealth of high resolution, GE-fMRI data that can be used for laminar analysis, but a lack of consensus on the optimal way to analyze these data, which could partially explain the variance in conclusions drawn from these data. By proposing robust yet simple analysis methods to remove the superficial bias, such as the use of Deming Regression, we hope these will help reconcile results across different studies, and allow researchers to probe deeper into the function of neuronal activity in different cortical layers.

## Data Availability Statement

The datasets presented in this study can be found in online repositories. The names of the repository/repositories and accession number(s) can be found below: https://github.com/MRC-CBU/LaminafMRIsimulations/releases/tag/1.2.

## Ethics Statement

The studies involving human participants were reviewed and approved by Cambridge Psychology Research Ethics Committee. The patients/participants provided their written informed consent to participate in this study. Written informed consent was obtained from the relevant individuals for the publication of any potentially identifiable images or data included in this article.

## Author Contributions

PH, MC, RH, and JC designed the experiments and analyzed the data. CR and CTR set up the acquisition protocols and assisted with data acquisition. PH wrote the manuscript. All authors reviewed the manuscript.

## Author Disclaimer

The views expressed here are those of the authors are not necessarily those of the NIHR or the Department of Health and Social Care.

## Conflict of Interest

The authors declare that the research was conducted in the absence of any commercial or financial relationships that could be construed as a potential conflict of interest.

## Publisher’s Note

All claims expressed in this article are solely those of the authors and do not necessarily represent those of their affiliated organizations, or those of the publisher, the editors and the reviewers. Any product that may be evaluated in this article, or claim that may be made by its manufacturer, is not guaranteed or endorsed by the publisher.

## Footnotes

1
www.fil.ion.ucl.ac.uk/spm

2
https://github.com/jooh/roitools/blob/master/spm2roi.m

3
https://github.com/MRC-CBU/LaminafMRIsimulations/tree/1.2

## Supplementary Material

Supplementary Figure 1 Plots of the simulated measured response **(A)** and attentional modulation metrics (Deming regression, Voxel ratio, ROI ratio, Z-scoring, L2 norm, SVM classification, and LDC, **B–F**) across different layers where voxels with the bottom 30% tSNR were excluded. These bars represent the median of all six participants, with each set of joint circles represent an individual subject. The same color represents the same participant throughout all plots. The axis range of panel **(B)** has been restricted due to extreme outliers for the voxel ratio. Note that these results are similar to that of Figure 7, suggesting that the exclusion of low tSNR voxels does not change the findings.

Supplementary Figure 2 Comparison of mean **(A)** and standard deviation **(B)** of t-statistic of the voxels in the ROI for real and simulated data. The error bars indicate the standard deviation of the summary metrics of the voxels across participants/iterations.

Supplementary Figure 3 Plots of the simulated response and attentional modulation metrics (Deming regression, Voxel ratio, ROI ratio, Z-scoring, L2 norm, SVM classification, and LDC) across different layers for different simulation parameters [reduced signal **(A)**, increased signal **(B)**, reduced noise **(C)**, and increased noise **(D)**]. The general trend is similar to that of the parameters used in the main paper (which were σ_face_ = 1.1, σ_house_ = 0.5, σ_phsiological_ = 11, and σ_thermal_ = 15). Key differences are that Deming regression becomes noisier with low signal or high noise while L2 normalization is able to remove the majority of superficial bias at high SNR.

Supplementary Figure 4 Plots of the attentional modulation metrics (Deming regression, Voxel ratio, ROI ratio, Z-scoring, L2 norm, SVM classification, and LDC) across different layers for the four ROIs [FFA **(A)**, OFA **(B)**, PPA **(C)**, and TOS **(D)**]. These bars represent the median of all six participants, with each set of joint circles represent an individual subject. The same color represents the same participant throughout all plots. Note that the results are similar to that of Figure 7, except with increase variance between participants. These results justify the pooling of the ROIs to reduce the impact of noise.

## References

[B1] Abdulkadir A. Ronneberger O. Christian Wolf R. Pfleiderer B. Saft C. Kloppel S. (2013). Functional and structural MRI biomarkers to detect pre-clinical neurodegeneration. *Curr. Alzheimer Res.* 10 125–134. 10.2174/1567205011310020002 22742852

[B2] Adcock R. J. (1878). A problem in least squares. *Analyst* 5 53–54. 10.2307/2635758

[B3] Alink A. Krugliak A. Walther A. Kriegeskorte N. (2013). fMRI orientation decoding in V1 does not require global maps or globally coherent orientation stimuli. *Front. Psychol.* 4:493. 10.3389/fpsyg.2013.00493 23964251 PMC3740242

[B4] Andersson J. L. R. Skare S. Ashburner J. (2003). How to correct susceptibility distortions in spin-echo echo-planar images: application to diffusion tensor imaging. *Neuroimage* 20 870–888. 10.1016/S1053-8119(03)00336-7 14568458

[B5] Arbuckle S. A. Yokoi A. Pruszynski J. A. Diedrichsen J. (2019). Stability of representational geometry across a wide range of fMRI activity levels. *Neuroimage* 186 155–163. 10.1016/j.neuroimage.2018.11.002 30395930

[B6] Bazin P. Weiss M. Dinse J. Schaefer A. Trampel R. Turner R. (2012). “A computational pipeline for subject-specific, ultra-high resolution cortical analysis at 7 Tesla,” in *Proceedings of the 18th Annu Meet Organ Hum Brain Mapp*, Bejing, 883.

[B7] Beckett A. J. Dadakova T. Townsend J. Huber L. Park S. Feinberg D. A. (2019). Comparison of BOLD and CBV using 3D EPI and 3D GRASE for cortical layer fMRI at 7T. *bioRxiv* [Preprint]. 10.1101/778142 PMC7721980 32557752

[B8] Boxerman J. L. Bandettini P. A. Kwong K. K. Baker J. R. Davis T. L. Rosen B. R. (1995). The intravascular contribution to fmri signal change: monte carlo modeling and diffusion-weighted studies in vivo. *Magn. Reson. Med.* 34 4–10. 10.1002/mrm.1910340103 7674897

[B9] Buxton R. B. Uludağ K. Dubowitz D. J. Liu T. T. (2004). Modeling the hemodynamic response to brain activation. *Neuroimage* 23 S220–S233. 10.1016/j.neuroimage.2004.07.013 15501093

[B10] de Hollander G. Van Der Zwaag W. Qian C. Zhang P. Knapen T. (2020). Ultra-high resolution fMRI reveals origins of feedforward and feedback activity within laminae of human ocular dominance columns. *bioRxiv* [Preprint]. 10.1101/2020.05.19.102186 33385565

[B11] De Martino F. Zimmermann J. Muckli L. Ugurbil K. Yacoub E. Goebel R. (2013). Cortical depth dependent functional responses in humans at 7T: improved specificity with 3D GRASE. Zhang N, editor. *PLoS One* 8:e60514. 10.1371/journal.pone.0060514 23533682 PMC3606277

[B12] Dumoulin S. O. Fracasso A. van der Zwaag W. Siero J. C. W. Petridou N. (2017). Ultra-high field MRI: advancing systems neuroscience towards mesoscopic human brain function. *Neuroimage.* 168 345–357. 10.1016/j.neuroimage.2017.01.028 28093360

[B13] Feinberg D. A. Vu A. T. Goebel R. Kemper V. G. Poser B. A. Yacoub E. (2015). Sub-millimeter T2 weighted fMRI at 7 T: comparison of 3D-GRASE and 2D SE-EPI. *Front. Neurosci.* 9:163. 10.3389/fnins.2015.00163 25999810 PMC4419681

[B14] Fischl B. Salat D. H. Busa E. Albert M. Dieterich M. Haselgrove C. (2002). Whole brain segmentation: automated labeling of neuroanatomical structures in the human brain. *Neuron* 33 341–355. 10.1016/S0896-6273(02)00569-X 11832223

[B15] Greve D. N. Fischl B. (2009). Accurate and robust brain image alignment using boundary-based registration. *Neuroimage* 48 63–72. 10.1016/j.neuroimage.2009.06.060 19573611 PMC2733527

[B16] Gudbjartsson H. Patz S. (1995). The rician distribution of noisy mri data. *Magn. Reson. Med.* 34 910–914. 10.1002/mrm.1910340618 8598820 PMC2254141

[B17] Havlicek M. Uludağ K. (2020). A dynamical model of the laminar BOLD response. *Neuroimage* 204:116209. 10.1016/j.neuroimage.2019.116209 31546051

[B18] Hoeft F. McCandliss B. D. Black J. M. Gantman A. Zakerani N. Hulme C. (2011). Neural systems predicting long-term outcome in dyslexia. *Proc. Natl. Acad. Sci. U.S.A.* 108 361–366. 10.1073/pnas.1008950108 21173250 PMC3017159

[B19] Huang P. Carlin J. D. Alink A. Kriegeskorte N. Henson R. N. Correia M. M. (2018). Prospective motion correction improves the sensitivity of fMRI pattern decoding. *Hum. Brain Mapp.* 39 4018–4031. 10.1002/hbm.24228 29885014 PMC6175330

[B20] Huang P. Carlin J. D. Henson R. N. Correia M. M. (2020). Improved motion correction of submillimetre 7T fMRI time series with boundary-based registration (BBR). *Neuroimage* 210:116542. 10.1016/j.neuroimage.2020.116542 31958583 PMC7068704

[B21] Huber L. (2020). Removing Unwanted Venous Signal From GE-BOLD Maps: Overview of Vein Removal Models and Implementations in LAYNII. Available online at: https://layerfmri.com/2020/04/02/devein/ (accessed August 2, 2020).

[B22] Huber L. Ivanov D. Hall A. Guidi M. Bandettini P. A. Gonzalez-Castillo J. (2017a). High-resolution CBV-fMRI allows mapping of laminar activity and connectivity of cortical input and output in human M1. *Neuron* 96 1253–1263.e7. 10.1016/j.neuron.2017.11.005 29224727 PMC5739950

[B23] Huber L. Uludağ K. Möller H. E. (2017b). Non-BOLD contrast for laminar fMRI in humans: CBF, CBV, and CMRO2. *Neuroimage* 197 742–760. 10.1016/j.neuroimage.2017.07.041 28736310 PMC12906290

[B24] Jia K. Zamboni E. Kemper V. Rua C. Goncalves N. R. Ng A. K. T. (2020). Recurrent processing drives perceptual plasticity. *Curr. Biol.* 30 4177–4187.e4. 10.1016/j.cub.2020.08.016 32888488 PMC7658806

[B25] Kamitani Y. Tong F. (2005). Decoding the visual and subjective contents of the human brain. *Nat. Neurosci.* 8 679–685. 10.1038/nn1444 15852014 PMC1808230

[B26] Kashyap S. Ivanov D. Havlicek M. Poser B. Uludag K. (2019). “Laminar CBF and BOLD fMRI in the human visual cortex using arterial spin labelling at 7T,” in *Proceedings of the 27th Scientific Meeting of ISMRM*, Montréal, QC, 609.

[B27] Kashyap S. Ivanov D. Havlicek M. Poser B. A. Uludağ K. (2017). Impact of acquisition and analysis strategies on cortical depth-dependent fMRI. *Neuroimage* 168 332–344. 10.1016/j.neuroimage.2017.05.022 28506874

[B28] Kay K. Jamison K. W. Vizioli L. Zhang R. Margalit E. Ugurbil K. (2019). A critical assessment of data quality and venous effects in sub-millimeter fMRI. *Neuroimage* 189 847–869. 10.1016/j.neuroimage.2019.02.006 30731246 PMC7737092

[B29] Kok P. Bains L. J. Van Mourik T. Norris D. G. De Lange F. P. (2016). Selective activation of the deep layers of the human primary visual cortex by top-down feedback. *Curr. Biol.* 26 371–376. 10.1016/j.cub.2015.12.038 26832438

[B30] Kriegeskorte N. Diedrichsen J. (2016). Inferring brain-computational mechanisms with models of activity measurements. *Philos. Trans. R. Soc. Lond. B Biol. Sci.* 371 489–495. 10.1098/rstb.2016.0278 27574316 PMC5003864

[B31] Lawrence S. J. Norris D. G. de Lange F. P. (2019). Dissociable laminar profiles of concurrent bottom-up and top-down modulation in the human visual cortex. *Elife* 8:e44422. 10.7554/eLife.44422 31063127 PMC6538372

[B32] Ledoit O. Wolf M. (2003). Improved estimation of the covariance matrix of stock returns with an application to portfolio selection. *J. Empir. Financ.* 10 603–621. 10.1016/S0927-5398(03)00007-0

[B33] Leprince Y. Poupon F. Delzescaux T. Hasboun D. Poupon C. Riviere D. (2015). “Combined Laplacian-equivolumic model for studying cortical lamination with ultra high field MRI (7 T),” in *Proceedings of the International Symposium on Biomedical Imaging (ISBI)*, Brooklyn, NY, 580–583. 10.1109/ISBI.2015.7163940

[B34] Liu C. Guo F. Qian C. Zhang Z. Sun K. Wang D. J. (2020). Layer-dependent multiplicative effects of spatial attention on contrast responses in human early visual cortex. *Prog. Neurobiol.* 101897. 10.1016/j.pneurobio.2020.101897 32818495

[B35] Liu T. T. (2016). Noise contributions to the fMRI signal: an overview. *Neuroimage* 143 141–151. 10.1016/j.neuroimage.2016.09.008 27612646

[B36] Lu H. Hua J. van Zijl P. C. M. (2013). Noninvasive functional imaging of cerebral blood volume with vascular-space-occupancy (VASO) MRI. *NMR Biomed.* 26 932–948. 10.1002/nbm.2905 23355392 PMC3659207

[B37] Markuerkiaga I. Barth M. Norris D. G. (2016). A cortical vascular model for examining the specificity of the laminar BOLD signal. *Neuroimage* 132 491–498. 10.1016/j.neuroimage.2016.02.073 26952195

[B38] Marques J. P. Kober T. Krueger G. van der Zwaag W. Van de Moortele P. F. Gruetter R. (2010). MP2RAGE, a self bias-field corrected sequence for improved segmentation and T1-mapping at high field. *Neuroimage* 49 1271–1281. 10.1016/j.neuroimage.2009.10.002 19819338

[B39] Meier T. B. Desphande A. S. Vergun S. Nair V. A. Song J. Biswal B. B. (2012). Support vector machine classification and characterization of age-related reorganization of functional brain networks. *Neuroimage* 60 601–613. 10.1016/j.neuroimage.2011.12.052 22227886 PMC3288439

[B40] Misaki M. Kim Y. Bandettini P. A. Kriegeskorte N. (2010). Comparison of multivariate classifiers and response normalizations for pattern-information fMRI. *Neuroimage* 53 103–118. 10.1016/j.neuroimage.2010.05.051 20580933 PMC2914143

[B41] Muckli L. De Martino F. Vizioli L. Petro L. S. Smith F. W. Ugurbil K. (2015). Contextual feedback to superficial layers of V1. *Curr. Biol.* 25 2690–2695. 10.1016/j.cub.2015.08.057 26441356 PMC4612466

[B42] Niazy R. K. De Stefano N. Beckmann C. F. De Luca M. Vickers J. Matthews P. M. (2004). Advances in functional and structural MR image analysis and implementation as FSL. *Neuroimage* 23 S208–S219. 10.1016/j.neuroimage.2004.07.051 15501092

[B43] Olman C. A. Inati S. Heeger D. J. (2007). The effect of large veins on spatial localization with GE BOLD at 3 T: displacement, not blurring. *Neuroimage* 34 1126–1135. 10.1016/j.neuroimage.2006.08.045 17157534

[B44] Olman C. A. Yacoub E. (2011). High-field fMRI for human applications: an overview of spatial resolution and signal specificity. *Open Neuroimag. J.* 5 74–89. 10.2174/1874440001105010074 22216080 PMC3245408

[B45] Petcharunpaisan S. (2010). Arterial spin labeling in neuroimaging. *World J. Radiol.* 2 384–398. 10.4329/wjr.v2.i10.384 21161024 PMC2999014

[B46] Polimeni J. R. Fischl B. Greve D. N. Wald L. L. (2010). Laminar analysis of 7T BOLD using an imposed spatial activation pattern in human V1. *Neuroimage* 52 1334–1346. 10.1016/j.neuroimage.2010.05.005 20460157 PMC3130346

[B47] Rockland K. S. (2017). What do we know about laminar connectivity? *Neuroimage* 197 772–784. 10.1016/j.neuroimage.2017.07.032 28729159

[B48] Rockland K. S. Pandya D. N. (1979). Laminar origins and terminations of cortical connections of the occipital lobe in the rhesus monkey. *Brain Res.* 179 3–20. 10.1016/0006-8993(79)90485-2 116716

[B49] Rua C. Costagli M. Symms M. R. Biagi L. Donatelli G. Cosottini M. (2017). Characterization of high-resolution Gradient Echo and Spin Echo EPI for fMRI in the human visual cortex at 7 T. *Magn. Reson. Imaging* 40 98–108. 10.1016/j.mri.2017.04.008 28438709

[B50] Stanley O. W. Kuurstra A. B. Klassen L. M. Menon R. S. Gati J. S. (2021). Effects of phase regression on high-resolution functional MRI of the primary visual cortex. *Neuroimage* 227:117631. 10.1016/j.neuroimage.2020.117631 33316391

[B51] Takahashi N. Oertner T. G. Hegemann P. Larkum M. E. (2016). Active cortical dendrites modulate perception. *Science* 354 1587–1590. 10.1126/science.aah6066 28008068

[B52] Uludağ K. Blinder P. (2018). Linking brain vascular physiology to hemodynamic response in ultra-high field MRI. *Neuroimage* 168 279–295. 10.1016/j.neuroimage.2017.02.063 28254456

[B53] Van Kerkoerle T. Self M. W. Roelfsema P. R. (2017). Layer-specificity in the effects of attention and working memory on activity in primary visual cortex. *Nat. Commun.* 8:13804. 10.1038/ncomms13804 28054544 PMC5227065

[B54] vonEconomo C. (1929). The Cytoarchitectonics of the Human Cerebral Cortex. *J. Anat.* 63(Pt 3):389.

[B55] Walther A. Nili H. Ejaz N. Alink A. Kriegeskorte N. Diedrichsen J. (2016). Reliability of dissimilarity measures for multi-voxel pattern analysis. *Neuroimage* 137 188–200. 10.1016/j.neuroimage.2015.12.012 26707889

[B56] Weygandt M. Blecker C. R. Schäfer A. Hackmack K. Haynes J. D. Vaitl D. (2012). FMRI pattern recognition in obsessive-compulsive disorder. *Neuroimage* 60 1186–1193. 10.1016/j.neuroimage.2012.01.064 22281674

[B57] Yoon J. H. Nguyen D. V. McVay L. M. Deramo P. Minzenberg M. J. Ragland J. D. (2012). Automated classification of fMRI during cognitive control identifies more severely disorganized subjects with schizophrenia. *Schizophr. Res.* 135 28–33. 10.1016/j.schres.2012.01.001 22277668 PMC3288252

[B58] Zamboni E. Kemper V. G. Goncalves N. R. Jia K. Karlaftis V. M. Bell S. J. (2020). Fine-scale computations for adaptive processing in the human brain. *Elife* 9 1–21. 10.7554/eLife.57637 33170124 PMC7688307

[B59] Zhu C. Z. Zang Y. F. Cao Q. J. Yan C. G. He Y. Jiang T. Z. (2008). Fisher discriminative analysis of resting-state brain function for attention-deficit/hyperactivity disorder. *Neuroimage* 40 110–120. 10.1016/j.neuroimage.2007.11.029 18191584

