# Supplementary figures and images for: Correcting for Superficial Bias in 7T Gradient Echo fMRI
Source: Front Neurosci. 2021 Sep 22;15:715549. doi: 10.3389/fnins.2021.715549 (PMC8494131; doi:10.3389/fnins.2021.715549)

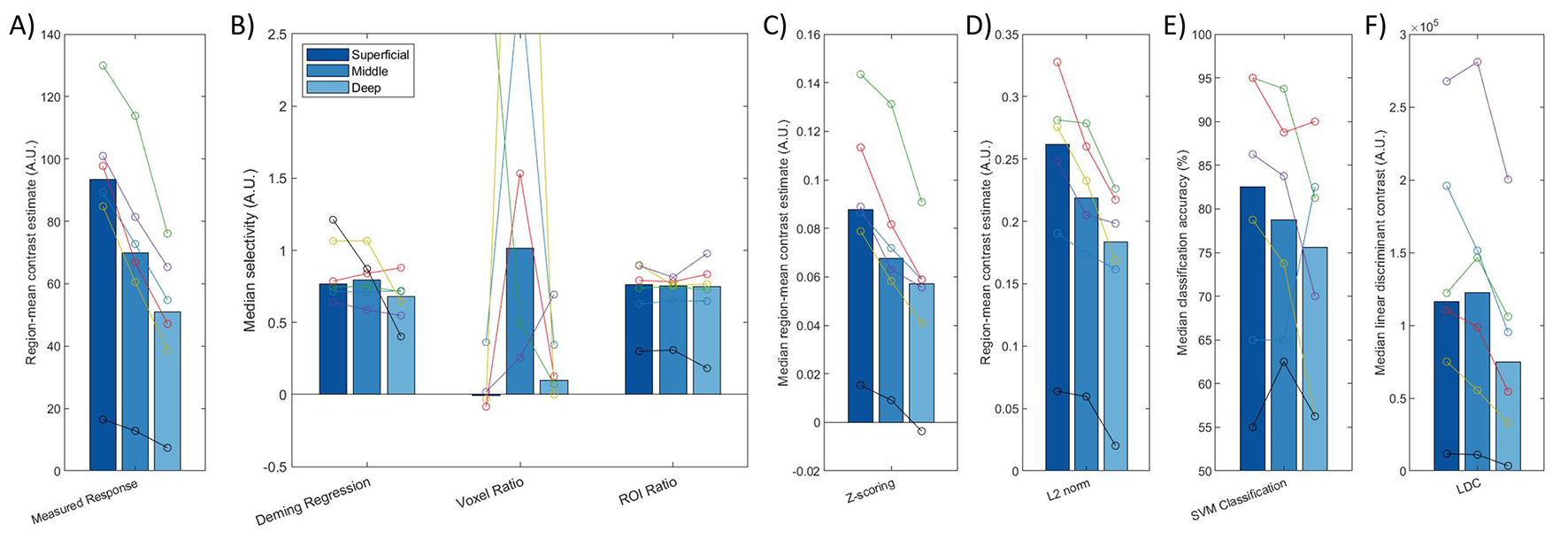

Supplement: Supplementary Figure 1 — Plots of the simulated measured response (A) and attentional modulation metrics (Deming regression, Voxel ratio, ROI ratio, Z-scoring, L2 norm, SVM classification, and LDC, B–F) across different layers where voxels with the bottom 30% tSNR were excluded. These bars represent the median of all six participants, with each set of joint circles represent an individual subject. The same color represents the same participant throughout all plots. The axis range of panel (B) has been restricted due to extreme outliers for the voxel ratio. Note that these results are similar to that of Figure 7, suggesting that the exclusion of low tSNR voxels does not change the findings. [file Image_1.JPEG]

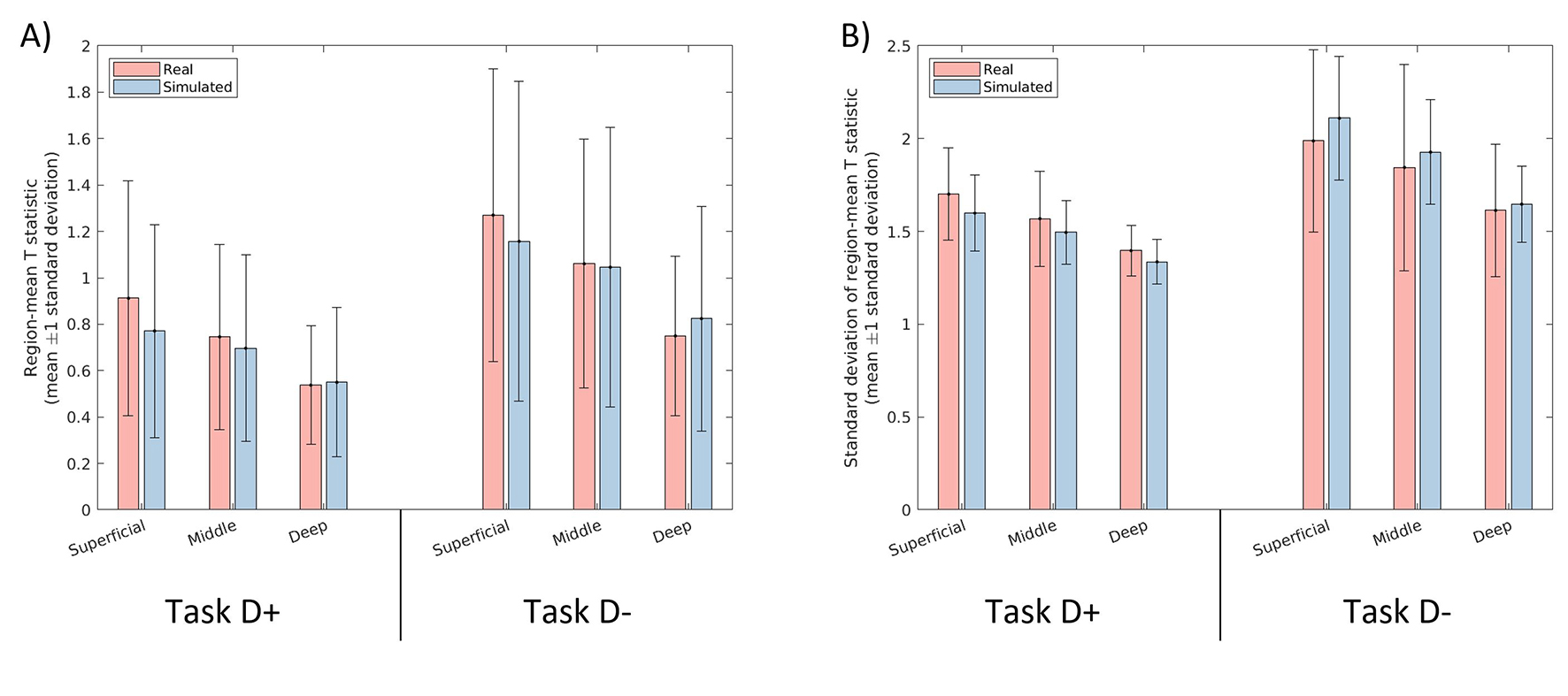

Supplement: Supplementary Figure 2 — Comparison of mean (A) and standard deviation (B) of t-statistic of the voxels in the ROI for real and simulated data. The error bars indicate the standard deviation of the summary metrics of the voxels across participants/iterations. [file Image_2.JPEG]

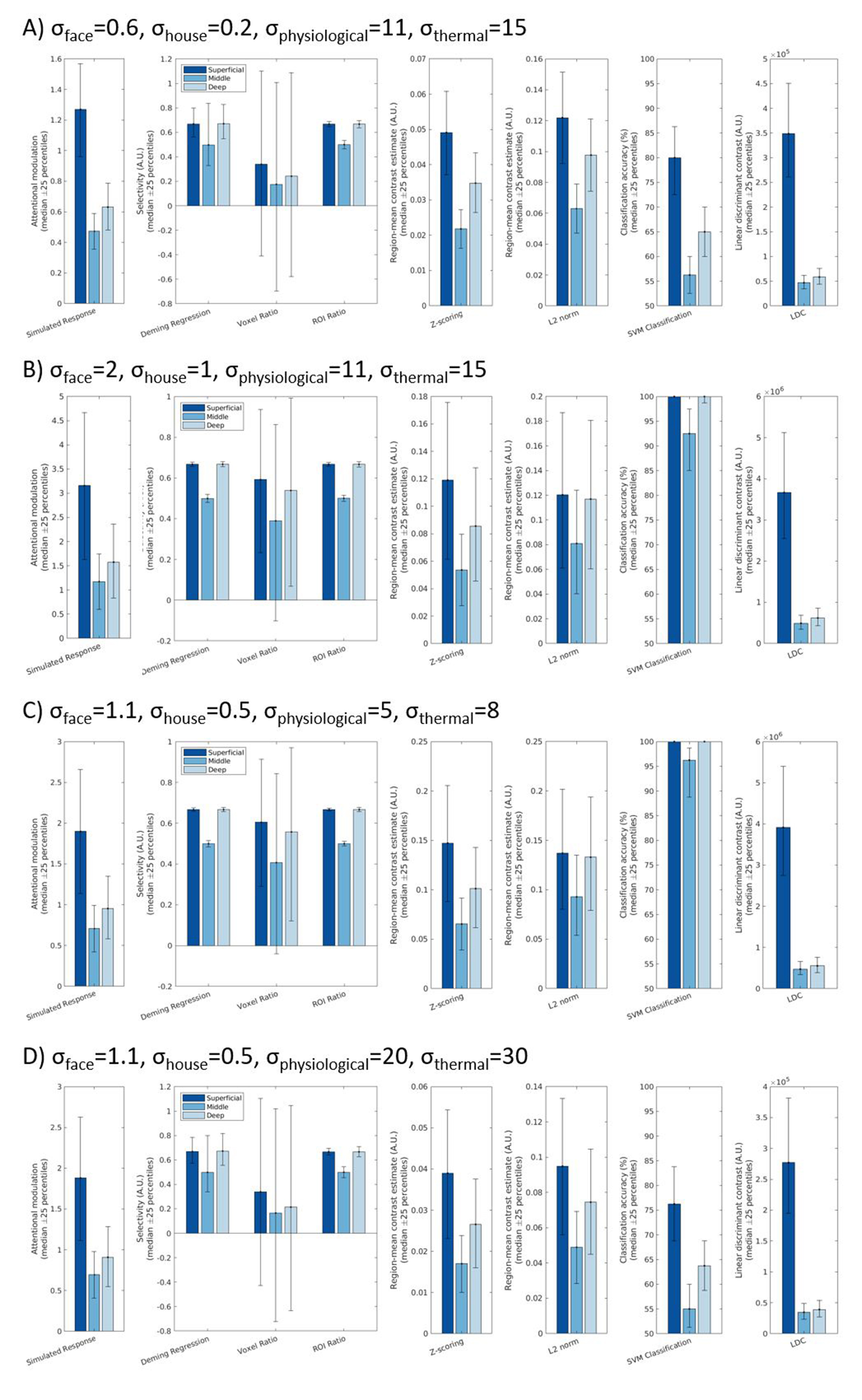

Supplement: Supplementary Figure 3 — Plots of the simulated response and attentional modulation metrics (Deming regression, Voxel ratio, ROI ratio, Z-scoring, L2 norm, SVM classification, and LDC) across different layers for different simulation parameters [reduced signal (A), increased signal (B), reduced noise (C), and increased noise (D)]. The general trend is similar to that of the parameters used in the main paper (which were σface = 1.1, σhouse = 0.5, σphsiological = 11, and σthermal = 15). Key differences are that Deming regression becomes noisier with low signal or high noise while L2 normalization is able to remove the majority of superficial bias at high SNR. [file Image_3.JPEG]

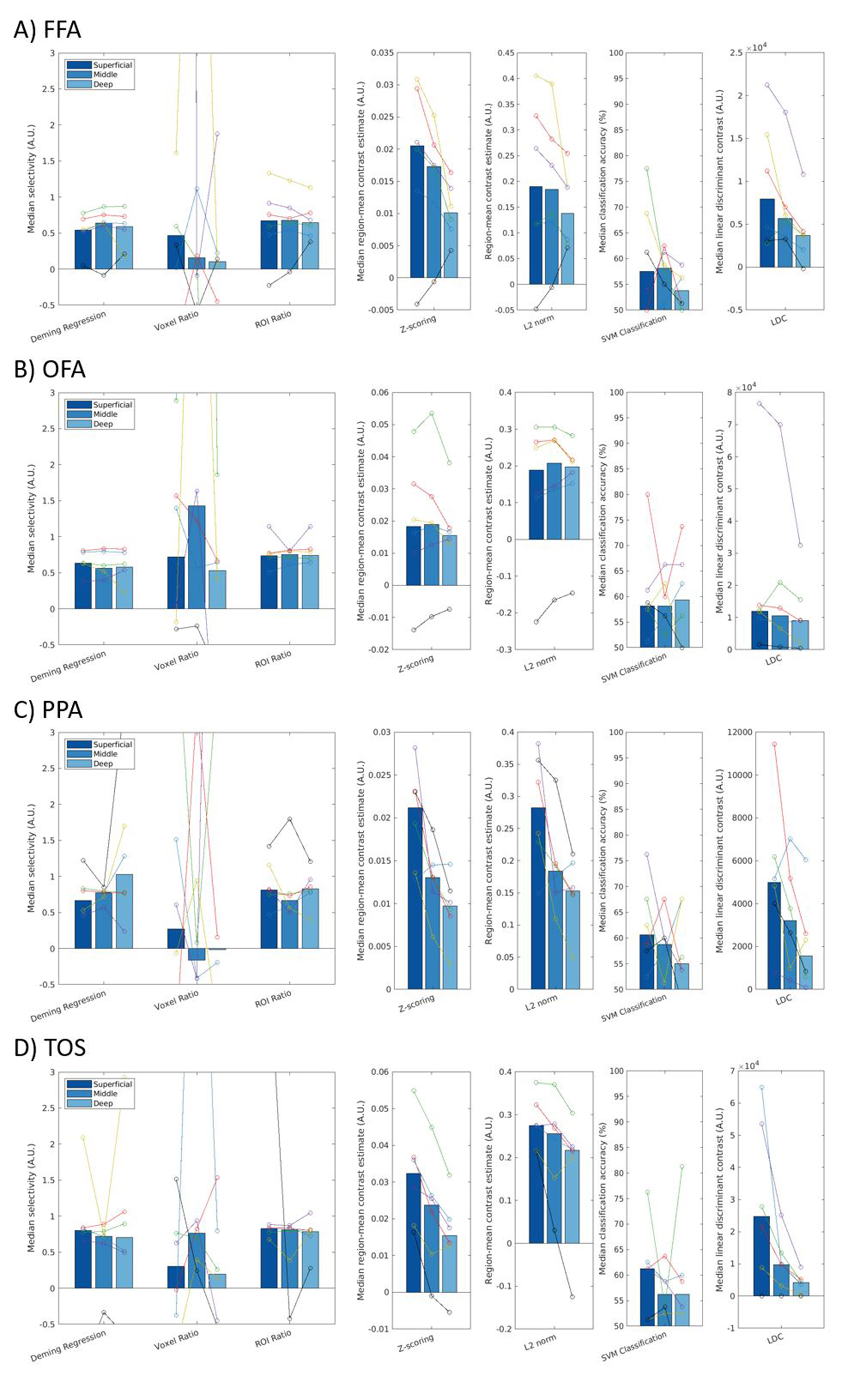

Supplement: Supplementary Figure 4 — Plots of the attentional modulation metrics (Deming regression, Voxel ratio, ROI ratio, Z-scoring, L2 norm, SVM classification, and LDC) across different layers for the four ROIs [FFA (A), OFA (B), PPA (C), and TOS (D)]. These bars represent the median of all six participants, with each set of joint circles represent an individual subject. The same color represents the same participant throughout all plots. Note that the results are similar to that of Figure 7, except with increase variance between participants. These results justify the pooling of the ROIs to reduce the impact of noise. [file Image_4.JPEG]
